# Towards Point-of-Care Single Biomolecule Detection Using Next Generation Portable Nanoplasmonic Biosensors: A Review

**DOI:** 10.3390/bios14120593

**Published:** 2024-12-04

**Authors:** Saeed Takaloo, Alexander H. Xu, Liena Zaidan, Mehrdad Irannejad, Mustafa Yavuz

**Affiliations:** 1 Department of Mechanical and Mechatronics Engineering, University of Waterloo, 200 University Ave. West, Waterloo, ON N2L 3G1, Canada; 2 Waterloo Institute for Nanotechnology (WIN), University of Waterloo, 200 University Ave. West, Waterloo, ON N2L 3G1, Canada; alexander.haoyan.xu@uwaterloo.ca (A.H.X.); lzaidan@uwaterloo.ca (L.Z.); 3 Department of Electrical and Computer Engineering, University of Waterloo, Waterloo, ON N2L 3G1, Canada; 4 OZ Optics Ltd., Ottawa, ON K0A 1L0, Canada; mirannejad@ozoptics.com

**Keywords:** nanoplasmonic, SPR, micro/nano-optics biosensor, plasmonic materials, point-of-care test, lab-on-a-chip

## Abstract

Over the past few years, nanoplasmonic biosensors have gained widespread interest for early diagnosis of diseases thanks to their simple design, low detection limit down to the biomolecule level, high sensitivity to even small molecules, cost-effectiveness, and potential for miniaturization, to name but a few benefits. These intrinsic natures of the technology make it the perfect solution for compact and portable designs that combine sampling, analysis, and measurement into a miniaturized chip. This review summarizes applications, theoretical modeling, and research on portable nanoplasmonic biosensor designs. In order to develop portable designs, three basic components have been miniaturized: light sources, plasmonic chips, and photodetectors. There are five types of portable designs: portable SPR, miniaturized components, flexible, wearable SERS-based, and microfluidic. The latter design also reduces diffusion times and allows small amounts of samples to be delivered near plasmonic chips. The properties of nanomaterials and nanostructures are also discussed, which have improved biosensor performance metrics. Researchers have also made progress in improving the reproducibility of these biosensors, which is a major obstacle to their commercialization. Furthermore, future trends will focus on enhancing performance metrics, optimizing biorecognition, addressing practical constraints, considering surface chemistry, and employing emerging technologies. In the foreseeable future, these trends will be merged to result in portable nanoplasmonic biosensors offering detection of even a single biomolecule.

## 1. Introduction

Early diagnosis of various diseases, including cancers, cardiovascular diseases, neurodegenerative disorders, etc., is significant in managing them because it can considerably improve treatment effectiveness, reduce healthcare costs, and often prevent illness progression [1,2,3]. A major obstacle to the early detection of these fatal diseases is the difficulty in detecting low levels of chemical biomarkers, even at the level of single biomolecules, using point-of-care testing (POCT) technologies [1,4]. To address the low concentration detection challenge, several optical biosensors, functioning based on photonic principles, have been designed and examined to detect a wide range of biomolecules and analytes. These optical technologies include plasmonic, fluorescence-based, surface-enhanced Raman scattering (SERS), interferometric, photonic crystal, whispering gallery mode, and colorimetric biosensors, to name but a few. The physical principle behind fluorescence-based biosensors is that they use fluorophore-labeled probes that emit light upon binding to targets. SERS works by amplifying Raman signals using nanomaterials. In photonic crystal biosensors, optical resonance shifts are monitored. Whispering gallery mode biosensors measure resonant light in microcavities. Colorimetric biosensors are designed to identify analytes by changing the color of a solution. The plasmonic biosensors discussed in this paper have attracted considerable attention due to their excellent ability to monitor light-matter interactions [5,6,7].

There have been five plasmonic processes reported in the literature [8,9], including surface plasmon resonance (SPR), plasmonic nanocavities, plasmon-induced resonance energy transfer, plasmon-enhanced absorption, and plasmonic SERS, which can be utilized for not only low-concentration but also single molecule detection. SPR biosensors measure changes in refractive index (RI) through absorption of biomolecules. Liedberg et al. introduced the SPR technique for biosensing applications in the initial work [10]. A silver metal film was used in SPR tests for gas measurement as a proof of concept. In their seminal paper in 1995 [11], IAsys and BIOS-1, two commercial SPR biosensors, were compared for applications in immunosensing and receptor-ligand research. The results of SPR tests demonstrated real-time and label-free studies of biomolecule interactions. Plasmonic nanocavities increase local field intensities due to their geometry and can be used in biosensors, optical trapping, and molecular studies [12]. Zhan et al. [13] introduced a plasmonic nanocavity capable of trapping and detecting single molecules (~2 nm) in solution and validated the results by experiments and simulations. Plasmon-enhanced absorption and plasmon-induced resonance energy transfer use plasmonic nanomaterials to amplify light absorption and boost energy transfer efficiency [8].

Following the promising results of SPR biosensors, nanoplasmonic biosensors (NPBs) have received remarkable interest owing to their high sensitivity to chemical adsorption, specificity, potential for multiplexing, low noise background, and ability to handle small sample volumes [14]. In addition, NPBs offer significant advantages due to their real-time monitoring features, small molecule detection, label-free detection, high reusability, short response times, and ease of operation and sample treatment [15,16,17]. The simplicity and cost-effectiveness of localized surface plasmon resonance (LSPR) biosensors have prompted their application in miniaturized, portable POCT devices [14]. Through the use of microfluidic technology, these biosensors are further enhanced by automating and multiplexing their analyses [18]. These features distinguish them from other conventional techniques, namely electrochemical, piezoelectric, and magnetic sensors, which may require more complex preparations and suffer from interference issues. For all limitations, including non-specific binding surfaces, mass transportation, and steric hindrance during binding events, there has been rapid progress in the development of plasmonic and NPBs.

This review paper aims to summarize research on portable nanoplasmonic biosensor designs. These NPB designs aim to provide high performance, cost efficiency, compactness, ease of use, and multiplexing sensing. These systems can also open up a wide range of applications, not least NPB POCT, that require portability. To achieve portability, bulky components, including light sources and photodetectors, have to be replaced with compact components. As opposed to Kretschmann-based SPR biosensors, nanoplasmonic designs are a more practical option, since oversized components are not needed. To do this, basic concepts in the field are reviewed to understand the plasmonic resonance phenomenon and the role of electromagnetic waves in this context. Basic concepts include the definition of the evanescent wave, plasmonic resonance condition, LSPR biosensors, hot spots of NPBs, main component of NPBs for portable design, and the most important biosensor characteristics, namely performance metrics. After understanding the basic concepts, the two most common dielectric models of plasmonic materials, as well as recently developed models, are presented, which is beneficial for future simulations of portable NPBs. Next, the literature on nanoparticles and nanostructures is reviewed. These studies allow us to gain insight into the design of plasmonic chips, the core components of NPBs. Following that, the literature on portable NPBs is reviewed, with a focus on integrating plasmonic components in portable NPBs. Remaining challenges and future trends for designing portable NPBs are reviewed in the final section to guide researchers towards future works.

## 2. Basic Concepts

Reviewing the literature on NPBs requires a basic understanding of elementary SPR theoretical concepts. SPR is defined as a phenomenon at the interface between specific materials, plasmonic materials, and dielectric materials. Surface plasmons are excited when polarized electromagnetic waves interact with the free electrons of plasmonic materials [19].

Surface plasmons are defined as the coherent oscillations of electrons generated at the interface of two materials. At this boundary, electrons oscillate because, in dielectric materials and metal (or plasmonic material), the sign of the dielectric function is opposite. Generally, dielectrics and conductive metals are the positive- and negative-permittivity materials, respectively. The oscillation of the charge leads to the emission of electromagnetic waves inside and outside the plasmonic material [19,20].

### 2.1. Evanescent Wave

**Figure 1:**
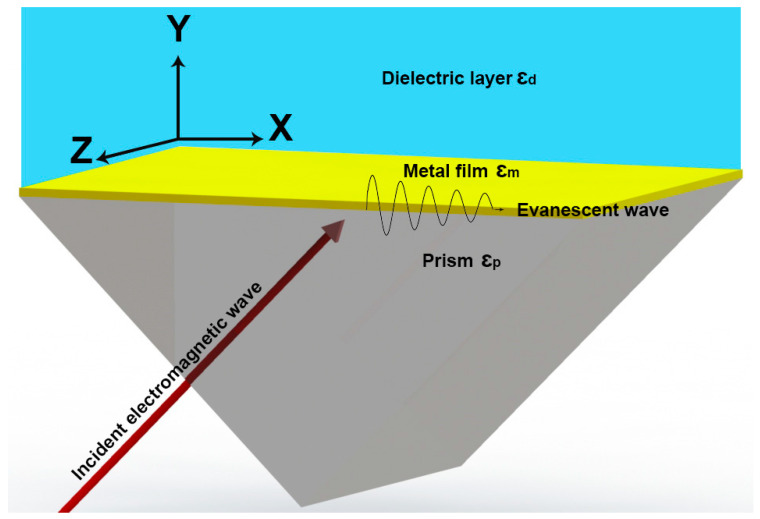
The schematic of SPR and evanescent waves. Evanescent waves occur at the metal-prism interface.

Evanescent waves are transverse electromagnetic waves propagating along materials’ interfaces. Technically, they are the result of internal reflection at the materials’ interfaces. According to Snell’s law, internal reflection occurs in the lower RI medium if the angle of the incident electromagnetic wave is larger than the critical angle. Figure 1 shows the schematic of SPR and the occurrence of evanescent waves. This wave propagates along the metal-prism interface.

Suppose that the incident electromagnetic wave, given in Equation (1), propagates from a higher RI (n1), prism, to a lower RI (n2), metal film. The evanescent waves, therefore, are generated in medium 2, denoted by electric field E_evn_ given in Equation (2). As shown in this mathematical model, the evanescent wave penetrates a short distance into the dielectric medium [21].

(1) $$E_{i}=E_{0}e^{j\left(\omega t-\mathrm{kr}\right)}=E_{0}e^{j\left(\omega t-k_{x}x-k_{y}y-k_{z}z\right)}$$

(2) $$E_{\mathrm{evn}}=E_{0}e^{{-k}_{y_{2}}y}e^{j\left(\omega t-k_{x}x\right)}$$

where E_0_ is the amplitude of the electric field, ω is the angular frequency, k is the wavevector, and r is the position vector. In these equations, k_x_ is the component of the wavevector along the propagation of the surface plasmon, while k_y_ is the component perpendicular to the surface. As can be seen, wavevector component k_x_ does not change, but the perpendicular component k_y_ changes. It can be inferred from Equation (2) that the strength of the electric field, E_evn_, decreases exponentially from the metal’s surface with a characteristic distance of 1/k_y2_. In practical applications, changes in the electric field far from SPR biosensors’ surface are not measurable. It is therefore necessary to use thin metal films in order to achieve proper SPR sensing.

### 2.2. Resonance Condition

SPR is the special case of surface plasmon excitation when the momentum of the incident electromagnetic wave matches the momentum of the surface plasmons. The momenta of the photons of electromagnetic waves increase in a prism as its refractive index increases. Equation (3) describes the wavevector of surface plasmons along an interface between a metal film and a dielectric [22]:

(3) $$k_{SPR}=\frac{\omega }{c}\sqrt{\frac{\varepsilon_{m}\varepsilon_{d}}{\varepsilon_{m}+\varepsilon_{d}}}$$

The wavevector of incident light along the metal-prism interface (k_x_) is equal to the wavevector of the surface plasmon wave (k_SPR_ in Equation (3)). In addition, there is a relationship between the light wavevector at the prism/metal film interface (k_x_) and the incident resonance angle. In Equation (4), this relationship is shown and the conditions for SPR are given [22]:

(4) $$k_{x}=\frac{\omega }{c}\sqrt{\varepsilon_{p}}\sin\theta_{\mathrm{res}}=\frac{\omega }{c}\sqrt{\frac{\varepsilon_{m}\varepsilon_{d}}{\varepsilon_{m}+\varepsilon_{d}}}=k_{SPR}\Rightarrow \sqrt{\varepsilon_{p}}\sin\theta_{\mathrm{res}}=\sqrt{\frac{\varepsilon_{m}\varepsilon_{d}}{\varepsilon_{m}+\varepsilon_{d}}}$$

where ɛ_p_, ɛ_m_, ɛ_d_, and θ_res_ are the dielectric constants of a prism, a metal film, the dielectric layer, and the incident resonance angle, respectively. It should also be noted that, as shown in Equation (4), SPR occurs not only at a specific angle (θ_res_), namely angular interrogation, but also at a specific wavelength (λ_res_), referred to as spectral interrogation. These parameters depend on the materials’ optical properties, and whether other molecules bind to the metal’s surface. Resonance conditions are highly sensitive to changes in the local RI, thereby allowing the molecules to be detected at the interface, namely the sensor surface, of the materials [21].

### 2.3. LSPR

LSPR involves the same physical principle as SPR, which is the oscillation of conduction electrons at the surface of a metal when excited by light. Additionally, in both sensing modes, shifts in the peak wavelength (Δλ) of plasmonic resonances are correlated with changes in RI (Δn) [23]. Their quantitative relationship is given in Equation (5).

(5) $$∆\lambda =m\Delta n\left(1-\exp\left(-\frac{2d}{l_{d}}\right)\right)$$

**Figure 2:**
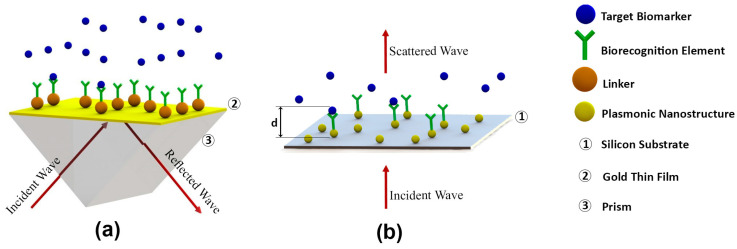
Comparison between SPR and LSPR biosensors (**a**) SPR biosensors (**b**) LSPR biosensors.

It should be noted that RI (n) can be obtained from the dielectric constant (ε), which is ε = n^2^. In Equation (5), m, d, and l_d_ represent the refractive index sensitivity, the effective adsorbate layer thickness, and the decay length of the electromagnetic field enhanced by nanostructures. According to [24], decay length l_d_ is calculated from the Maxwell’s equations. Additionally, the parameter d is defined schematically in Figure 2b.

Besides the peak wavelength value, the extinction intensity can also be used as a sensing signal. The reason for this is that the extinction intensity of an analyte near a metallic surface changes depending on its concentration. The development of LSPR biosensors based on intensity is very important since such sensors are cheaper, smaller, and easier to integrate into miniaturized, portable devices than spectrometers [25,26].

While plasmon resonance in both LSPR and SPR involves the aforementioned similarities, they also have distinct differences. In contrast to SPR mode, which occurs on relatively large metal surfaces, LSPR mode occurs in the vicinity of nanostructures, including nanoparticles [27], nanoholes [28], nanowires [29,30], and nanorods [31]. In other words, the electromagnetic field is enhanced on the electron clouds of nanostructures in LSPR. In terms of resonance conditions, the LSPR wavelength depends on the size, shape, and material of the nanoparticles, as well as the dielectric properties of the surrounding medium of the nanostructure [32]. In Figure 2, SPR and LSPR biosensors are schematically compared.

### 2.4. Hot Spots

The term “hot spot” refers to regions within nanostructures where electromagnetic fields are intensely enhanced and highly sensitive to changes in dielectric properties within the decay length of an evanescent wave. Hot spots can be created mainly by designing a single nanoparticle’s shape, i.e., the tips of nanoparticles, and arrays of nanostructures, i.e., the narrow space between two nanostructures. Accordingly, the theoretical analysis of electromagnetic field distribution around a nanostructure through finite-difference time-domain (FDTD) simulations can be used to determine whether the nanostructures are suitable for plasmonic sensing [33,34].

### 2.5. Main Components

SPR biosensors typically consist of a plasmonic chip, light source, polarizer, wave detector, and analyzer electronics board. The plasmonic chip, the core component of the device, responds to the target analyte via the SPR mechanism. Functionalizing nanostructures with biological recognition elements in LSPR biosensors makes the device sensitive to target biomarkers, subsequently resulting in a blue or red shift in the recorded resonance wavelength of the nanostructure. The wave source is intended to produce electromagnetic waves with wavelengths in the range of plasmonic operation. The polarizer is a device to transmit polarized waves, which is essential for proper biosensor operation. The wave detector device and analyzer electronic board are intended to analyze the reflected wave at the sensor’s surface and find the plasmonic peak wavelength. In Figure 2, the schematics of the components of SPR and LSPR biosensors are also shown [35,36]. Current research has employed portable spectrometers to perform integrated light source, polarizer, and wave detector measurements.

In addition to conventional detection techniques, the integration of Surface-Enhanced Raman Spectroscopy (SERS) with LSPR in biosensors has been widely studied [37]. This is because advancements in LSPR and SERS techniques have led to the development of highly sensitive NPBs for the detection of many types of diseases, not least cancers [38]. Besides the advantages of using SERS, serious limitations, including the difficulty of creating uniform and reproducible hot spots, biocompatibility, durability, large heat generation, and easy oxidization, exist [39]. Gabudean et al., in [40], studied the adsorption of p-aminothiophenol on gold nanorods using LSPR and SERS spectroscopies, and they evaluated the origin of electromagnetic enhancement in SERS. The results showed that electromagnetic enhancement occurs at the ends of gold nanorods and between coupled rods where the sensitive detection of molecules is feasible. An LSPR-SERS plasmonic sensor based on triangular silver nanoparticles coated with chitosan was reported by Potara et al. in [41] for the detection of the adenine molecule. The use of gold nanocylinder arrays as plasmonic materials in the detection of bovine serum albumin and ribonuclease-A using a SERS technique has been studied in [42]. Their results showed that optimized SERS is efficient for reproducible NPB.

### 2.6. Performance Metrics

To assess and compare the performance of biosensors, some indicator parameters are used, including sensitivity, limit of detection (LOD), accuracy, specificity, dynamic range, and reproducibility. The sensitivity (S) of NPB is defined by Equation (6). In this equation, “A” represents the shift in peak wavelength and its unit is nanometers. The parameter “n” is the RI, and its unit of measurement is the refractive index unit (RIU). This parameter can range from 50 nm/RIU to 30,000 nm/RIU depending on the designed structures and materials [43,44]. Biosensor accuracy shows the deviation between a measured and a true value. Reproducibility refers to the ability of a fabrication technique to provide a stable output under the same conditions. Dynamic range refers to the measurement range of biosensors.

(6) $$S=\frac{∆A}{∆n}$$

LOD represents the smallest quantity of change detectable by the system, as defined in Equation (7), where m is a numerical factor (typically 2 or 3), and σ_blank_ is the standard deviation of the blank measures [43]. LOD and σ_blank_ have the same unit as concentration (fg/mL, ng/mL, etc.). In addition, the parameters m and S have the same unit, nm/RIU. Based on Equation (7), reducing the noise of detectors and sources will obviously improve LOD. This has been the focus of previous works. Jin et al. have suggested a methodology based on an upconverted detection system, which potentially suppresses sampling noise level in a CMOS-compatible heterodyne interferometer by two orders of magnitude [45]. The results of the study showed that single polystyrene nanobeads and HIV1 virus-like particles could be detected with LOD down to a few attogram levels. Therefore, new techniques could be employed to improve biosensor LOD.

(7) $$\mathrm{LOD}=\frac{m\sigma_{\mathrm{blank}}}{S}$$

In addition to these above common indicators of optical biosensors, there are two other significant parameters for NPB, namely full width at half maximum (FWHM) and figure of merit (FOM). FWHM refers to the width of the resonance peak at half-intensity of its maximum value, and its unit is nanometers. The second parameter, figure of merit (FOM), defined in Equation (8), is the efficiency of biosensors to measure small RI changes, and its unit is RIU^−1^. The larger FOM, therefore, is desirable. In contrast, a high FWHM is considered a negative factor in plasmonic biosensing. In recent years, there has been a growing interest in studying how materials and nanostructures influence the FWHM and FOM of NPB. The FWHM of biosensors depends upon several factors, including the shape of the nanostructure, its material, the dielectric environment, and its peak wavelength [44].

(8) $$\mathrm{FOM}=\frac{S}{\mathrm{FWHM}}$$

## 3. Theoretical Modeling of Plasmonic Resonance

Plasmonic resonance phenomena are theoretically explained in terms of the dispersion relation of surface plasmonics and the dielectric function of the material. In a material, the dielectric function refers to the response of free and bound electrons to an external electric field. In order for SPR to occur, the dispersion relation must be satisfied, which is the matching of the wavevector of the incident light to the SPR wave.

### 3.1. Dielectric Models

The dielectric function of a material (*ϵ*(*ω*) = *ϵ*_real_(*ω*) + i *ϵ*_imag_(*ω*)) describes the interaction between its free electrons and an electromagnetic field. The real part of the dielectric function, *ϵ*_real_(*ω*), represents the material’s dispersive properties, which determine the phase velocity of light as it propagates in the material. It also gives information about the material’s ability to store energy from the electromagnetic field in the form of polarization. The imaginary part of the dielectric function, *ϵ*_imag_(*ω*), is related to the material’s absorptive properties [46]. The Drude model and the Lorentz model are two models commonly used to model the dielectric function.

#### 3.1.1. Drude Model

In the Drude model, conduction electrons are modeled as a gas of free particles around ion cores [47]. The model is derived from ideal gas laws and is relatively accurate for metals. In this model, Drude has made three main assumptions, namely free electron, independent electron, and relaxation time. According to the free electron assumption, electron gas interactions with ion cores are ignored and considered as particle interactions. It is also assumed that the interactions between electrons in the gas are ignored based on the second assumption, the independent electron. The relaxation time estimation refers to the average amount of time an electron spends moving through a medium before colliding with another electron. It is also important to note that the Drude model does not accurately estimate all real metals [48]. Based on these simplifying assumptions, Drude’s complex dielectric function is expressed as follows:

(9) $$\epsilon_{r}\left(\omega \right)=\epsilon_{r,\infty }\left(1\;-\frac{\omega_{p}^{2}}{\omega \left(\omega +i\gamma_{P}\right)}\right)$$

where ɛ_r, ∞,_ ω_p_, and γ_p_ are the dielectric constant at high frequencies, the plasma frequency of oscillation for the free electron gas, and the damping coefficient, respectively.

#### 3.1.2. Drude-Lorentz Model

The Lorentz model is a modification of Drude model, which removes the independent electron assumption [49]. While this assumption is relatively accurate at optical frequencies, it is the main cause of the differences between Drude model results and actual measurements. To overcome this limitation, the electron-ion interaction and electromagnetic field are modeled as two masses attached by a spring and the driving force in the mechanical system, respectively. A third term is added to the dielectric function, namely the Lorentz oscillator term [48]. Considering this assumption, the modified Drude-Lorentz dielectric function model is formulated as follows:

(10) $$\epsilon_{r}\left(\omega \right)=\epsilon_{r,\infty }\left(1-\frac{\omega_{p}^{2}}{\omega \left(\omega +i\gamma_{P}\right)}\right)+\sum_{j=1}^{M}\frac{f_{j}\omega_{p,j}^{2}}{\omega_{j}^{2}-\omega^{2}+i\omega \gamma_{j}}$$

where f_j_ and ω_0,j_ are the strength of the *j*-th oscillator and the resonant frequency of *j*-th oscillator, respectively.

#### 3.1.3. Recent Dielectric Models

Beside the Drude and Drude-Lorentz models, other dielectric function models for materials have been reported [46]. Recent models have been developed based on previous Drude models [50]. In the literature, the Brendel-Bormann model has been reported widely, which features improved fitting accuracy [51]. In this model, which is shown in Equation (11), a Gaussian integration is included in each Lorentz oscillator, which has made the optical spectra prediction more accurate. In addition, the multiple oscillator model, derived from the Lorentz model, has been widely used in the literature to accurately predict the dielectric function in the infrared and visible spectral ranges [52]. In the other study by Prokopeva et al. [53], a generalized dispersive material model based on Pade approximants was presented, and its approximation accuracy was compared to the classical recursive-convolution technique. These models accurately predict the dielectric properties of the alloys and have been validated with experimental results.

(11) $$\epsilon_{r}\left(\omega \right)=\epsilon_{r,\infty }\left(1-\frac{\omega_{p}^{2}}{\omega \left(\omega +i\gamma_{P}\right)}\right)+\sum_{j=1}^{M}\frac{1}{\sqrt{2\pi }\sigma_{j}}\int \exp\left[-\frac{{\left(x-\omega_{j}\right)}^{2}}{2\sigma_{j}^{2}}\right]\frac{f_{j}\omega_{p,j}^{2}}{\omega_{j}^{2}-\omega^{2}+i\omega \gamma_{j}}\mathrm{dx}$$

While the Drude and Drude-Lorentz models could be used in many applications, the newly developed models are more accurate in particular materials. For instance, in [54], the dielectric functions of gold and silver were modeled in both bulk and nanometric states, using various theoretical models, including the Drude Lorentz theory, the Drude two-point critical model, and the Drude three-point critical model. Rioux et al. [55] reported the genetic algorithm-fitted, multi-parametric analytical model, derived from the Drude-Lorentz model for gold–silver alloys. In [56], Derkachova et al. presented a dielectric function model for gold that addresses interband transitions above the energy threshold of 1.8 eV and small size effects. The results of these models were validated by the experimental data for gold monodisperse colloidal nanospheres, and the literature. As a result, modeling and re-tuning the dielectric function model for specific metals and wavelengths will improve the accuracy of the simulation.

These models have been compared in the literature. Jahanshahi et al., in [50], reported that, among all models for SPR sensing, the Brendel-Bormann model is the most accurate for determining permittivity values. They also considered the impact of a titanium layer used to fix gold thin films. Vial and Laroche, in [57], showed that combining Drude and critical point models accurately estimates the dielectric function of various metals for FDTD simulations. They also found that a thin chromium layer causes resonance blue-shifts, broadens FWHM, reduces field enhancement, and should be considered in optimizing structures for accurate predictions. These models are also implemented in time-domain simulations, including FDTD and auxiliary differential equations (ADE) [58,59]. Therefore, while great effort has been devoted to modeling dielectric functions accurately, more research is needed in this field.

### 3.2. Dispersion Relation

Since the SPR wave is a longitudinal wave along the materials interface, the momentum of the incident photon and the SPR wave are the same. The dispersion relation represents the propagation constant of the surface plasma wave propagating at the interface between a semi-infinite dielectric and metal, which is given by Equation (11). According to this equation, the SPR occurs if the metal has a negative real and small positive imaginary dielectric constant, which is the same case as with gold and silver. This equation is derived from Maxwell’s partial differential equations.

(12) $$k\left(\omega \right)=\frac{\omega }{c}\sqrt{\frac{\epsilon_{m}\epsilon_{d}}{\epsilon_{m}+\epsilon_{d}}}=\frac{\omega }{c}n_{p}\sin\left(\theta_{i}\right)$$

where k(ω) is the propagating constant of surface plasmons, ɛ is the relative permittivity of the materials, namely the prism (ɛ_d_) and the metal film (ɛ_m_), ω is the angular frequency of the incident EM wave, n_p_ is the refractive index of the coupling prism, and c is the speed of light in a vacuum.

## 4. Nanoparticles and Nanostructures

The controlled design and fabrication of plasmonic materials can have a significant impact on their optical properties. It has been demonstrated that the size, shape, and composition of plasmonic materials affect the resonance shifts of the plasmonic materials [60,61]. As a result of size and shape tunability, highly sensitive and selective biosensors are possible, which are capable of detecting target molecules without interference from other biological components [62]. Additionally, in the literature, the optical properties of nanofilms and nanoparticles have also been studied significantly [62]. Recent advances in material science have led to the exploration of alternative plasmonic materials. Metals such as gold (Au), silver (Ag), and copper (Cu), doped semiconductors such as aluminum-doped zinc oxide (AZO) and indium-doped tin oxide (In−SnO_2_), and dielectrics such as titanium nitride (TiN) make up the majority of plasmonic materials [63]. Doped semiconductors and dielectrics are more chemically stable and tunable than traditional metal-based plasmonic materials [63]. Because metals have an abundance of free electrons, they are generally suitable for plasmonic applications [64], but they have certain limitations, including limited chemical stability, high ohmic losses, and non-tunable conductivity. Doping semiconductors, on the other hand, results in the formation of surface plasmons and two-dimensional (2D) nanomaterials, such as graphene, which support surface plasmons [65,66,67].

Two-dimensional nanomaterials have attracted significant interest for their potential plasmonic applications. Graphene, for instance, exhibits an impressive level of tunability in both its electronic and optical properties, which can be harnessed for the development of efficient plasmonic devices operating at terahertz to mid-infrared frequencies [66,67,68]. The unique properties of 2D nanomaterials coupled with novel fabrication techniques can be used to design and optimize plasmonic devices for biosensing applications that have better performance, high sensitivity, and high selectivity.

The integration of nanoparticles provides several advantages, including increased mechanical strength, higher heating rates, and prevention of AuNP aggregation [69]. For instance, Duan et al., in [70], used chitosan-stabilized gold nanoparticles to decorate graphitic carbon nitride (g-CN) sheets and MoS2 quantum dots. MoS2 and g-CN were prepared by exfoliation, while the gold nanoparticles were prepared by PECVD. A key challenge in the integration of 2D materials with metallic nanoparticles is the precise control over nanoparticle distribution and size on the surface of the 2D material. The issue has been addressed in a variety of ways, including chemical vapor deposition, self-assembly, and wet-chemical methods. For example, nanoparticle arrays on 2D materials have been created using Langmuir-Blodgett techniques and block copolymer-assisted self-assembly. Furthermore, the functionalization of 2D materials with specific ligands or surfactants can facilitate the controlled growth and attachment of metallic nanoparticles. For tailoring the hybrid system’s desired properties, precise control over nanoparticle integration is crucial, since it impacts plasmonic response, charge transfer, and catalytic activity. As a result of interactions between 2D materials and metallic nanoparticles, new phenomena and emergent properties may arise, such as plasmon-exciton coupling, surface-enhanced Raman scattering, and hot electron generation, which can be exploited for various applications in sensing, energy conversion, and photonic devices [71,72]. Table 1 presents a brief overview of plasmonic nanoparticles and nanostructures, their merits and limitations.

## 5. Portable NPBs

Recently, portable designs for NPBs have gained significant attention in the literature because of their numerous advantages, including on-site testing, rapid decision-making, and use in rural health clinics. Employing portable POCT biosensors in remote locations eliminates the need for sending samples to laboratories and waiting for results [73]. Moreover, rapid results in medical applications can facilitate rapid medical treatment, which can stop acute medical conditions from getting worse. Developing portable NPBs, therefore, requires a miniaturized, low-cost, easy-to-use for non-expert operation, and lightweight design [74]. Current advances in nanoparticles and nanostructures have also facilitated portable NPB designs. It has been demonstrated that integrating 2D materials with metals and semiconductors enhances NPB performance [75]. We review the research that addresses the problem of designing portable NPBs in this section.

**Figure 3:**
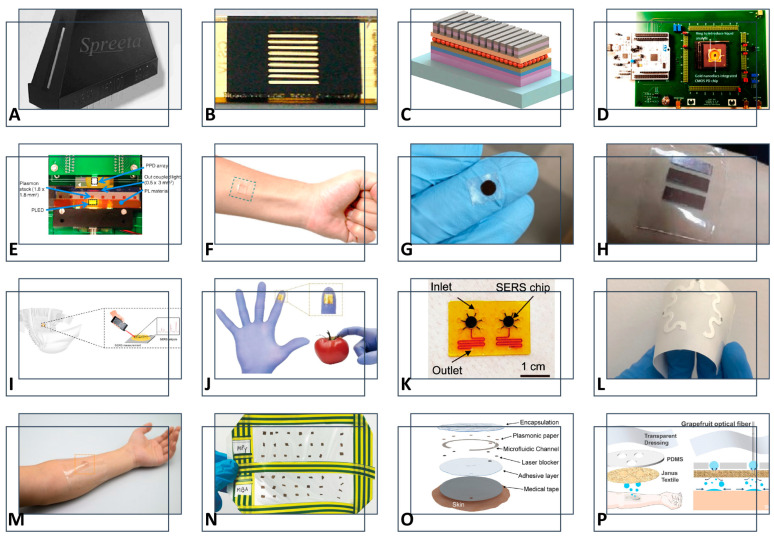
Portable NPBs, including portable SPR, miniaturization SPR components, flexible NPBs, wearable SERS-based designs, and microfluidic NPBs. (**A**) Spreeta 2000 plasmonic sensor, reproduced with permission. Ref. [76], copyright 2003, Elsevier. (**B**) Gold-coated diffraction grating sensor chip integrated with microfluidics, reproduced with permission. Ref. [77], copyright 2010, Elsevier. (**C**) Integrated quantum dot LED with plasmonic nanograting, reproduced with permission. Ref. [78], copyright 2015, IOP Publishing Ltd. (**D**) Integration of array of gold nanodiscs to CMOS chip, reproduced with permission. Ref. [79], copyright 2016, American Chemical Society. (**E**) Polymer integrated biosensing system, reproduced with permission. Ref. [83], copyright 2010, John Wiley & Sons. (**F**) Flexible LSPR biosensor based on AuNP/APTES/PDMS. reproduced with permission. Ref. [80], under the CC-BY 4.0 license. (**G**) Wearable SERS biosensors based on gold nanostars for sweat monitoring. reproduced with permission. Ref. [81], under the CC-BY 4.0 license. (**H**) Wearable SERS Sensor Based on Omnidirectional Plasmonic Nanovoids Array, reproduced with permission. Ref. [84], copyright 2022, John Wiley & Sons. (**I**) Wearable diaper sensor and handheld Raman spectrometer for urinalysis, reproduced with permission. Ref. [85], copyright 2003, Elsevier. (**J**) Ultrathin wearable 3D particle-in-cavity SF-AAO-Au SERS biosensors for glucose and pesticide detection, reproduced with permission. Ref. [86], copyright 2022, Elsevier. (**K**) Wearable microfluidic nanoplasmonic biosensor based on a miniature, thin plasmonic metasurface with homogeneous mushroom-shaped hot spots and high SERS activity, reproduced with permission. Ref. [87], under the CC-BY 4.0 license. (**L**) Wearable SERS–based microfluidic system for sweat rate and sweat lose quantification, reproduced with permission. Ref. [88], under the CC-BY 4.0 license. (**M**) Wearable and Flexible plasmonic biosensor based on SERS fingerprint analysis of chemical biomarkers, reproduced with permission. Ref. [89], copyright 2022, Science Advances. (**N**) Wearable flexible SERS biosensors based on Au/TPU nanofibers, reprinted with permission from [90]. Copyright 2021 American Chemical Society. (**O**) Flexible wearable plasmonic paper-based microfluidic SERS biosensor based on, reprinted with permission from [91]. Copyright 2024 American Chemical Society. (**P**) wearable Janus fabric/grapefruit optical fiber embedded with Ag nanoparticles, reprinted with permission from [92]. Copyright 2024 American Chemical Society.

The comparison of the existing portable NPB designs in the literature is shown in Figure 3. Portable NPBs integrate advanced materials and sensing technologies for enhanced sensitivity and flexibility. Spreeta 2000 (Figure 3A, [76]) and gold-coated diffraction grating systems (Figure 3B, [77]) are two examples of designing portable SPR biosensors. In contrast to previous SPR technologies, recent research has addressed the problem of miniaturization of optical components, because all components are required to be miniaturized to design portable NPBs. Two representatives of this research are shown in Figure 3C,D: quantum dot LED [78] and CMOS-integrated sensors [79]. Three primary components are needed to achieve the basic functionality of an NPB: a light source that stimulates the surface plasmonic wave, a sensing device that interacts with the analytes, and a detector that observes the propagation of the wave following the interaction. In addition, flexible substrates (Figure 3F, [80]) for LSPR sensing have been examined for developing lightweight NPBs. A particularly exciting development is the rise in wearable SERS-based biosensors (Figure 3G, [81]), which employ gold nanostructures, flexible substrates, and microfluidic systems. Microfluidics has enabled the development of NPBs that can manipulate nanoliters and picoliters of analytes close to the sensing device [82]. In the following subsections, recent progress in these five groups of portable NPBs, namely portable SPR, miniaturization components, flexible NPBs, wearable SERS-based designs, and microfluidic NPBs, is discussed.

### 5.1. Portable SPR

Kretschmann configurations are commonly used in portable SPR designs because of their high sensitivity and efficient coupling. Chinowsky et al. in [76] presented the Spreeta 2000 portable SPR, which included an LED light source, a plastic prism for phase matching, a gold film for sensing, and a linear array of diodes for detection (Figure 3A). Additionally, the device included a polarizer for focusing light and a memory chip for storing information. To prove its sensing functionality, an array of mouse immunoglobulin G proteins and protein A, a bacterial cell wall protein, was used. The protein–protein interactions were measured by Spreeta 2000, producing quantitative data describing these interactions with great accuracy. The overall dimensions of the device were 1.5 cm × 0.7 cm × 3 cm in the latest model. This device has the advantage of integrating all biosensing components into a compact package. There is, however, no monolithic integration, and the prism increases the size of the sensor. The LED source’s integration is advantageous in terms of size and cost, but it also requires a polarizer to ensure a more focused signal, which contributes to the sensor’s enlarged size. In a later version, microfluidic channels were incorporated to enhance performance by creating an injection loop flow system for optimal sensing [93].

A slightly larger plasmonic biosensor measuring 2.4 cm × 0.8 cm × 4.5 cm was described in [94], with multiple inlets and outlets for multiplexed sensing of a toxin, 2,4-dichlorophenoxyacetic acid (2,4-D), and a microfluidic channel for improved analyte manipulation. The detection limit of this device is 0.1 ppb, which is equivalent to 0.1 ng/mL. However, the prism increases the sensor’s cost. A rotating mirror generates monochromatic light in [95] without using any lenses. Despite this, it is difficult to synchronize the mirror rotation rate with the frame rate of a charge-coupled device (CCD) camera. A CCD camera allows for simultaneous and real-time imaging of light intensity at different locations, allowing for simultaneous detection of multiple analytes and, therefore, increased throughput [82].

In [96], a biosensing platform for pathogen detection utilizing a thin gold film and the Kretschmann configuration was presented. For light detection, a complementary metal-oxide-semiconductor (CMOS) sensor was used in conjunction with poly(methyl methacrylate) (PMMA) microfluidic channels for the flow of Escherichia coli solution. In comparison to other commercial devices, the presented system is portable, lightweight, and employs an economical fabrication process. However, due to the platform’s high limit of detection of about 10^5^ CFUs/mL, it is not suitable for sensing analytes at low concentrations. Because the system used an external LED as the light source and a lens for focusing the light, it was not entirely miniaturized. The study [97] introduced a very similar Kretschmann platform for blood protein detection. Although Kretschmann’s SPR technology was simple and reliable, miniaturization was limited, so recent research has focused on LSPR-based portable devices.

### 5.2. Miniaturization of Optical Components

#### 5.2.1. Light Sources

Despite considerable research on compact and portable plasmonic biosensors, achieving complete miniaturization and integration remains a challenge. For obtaining a focused monochromatic wave to interact with the sensing surface, lenses and polarizers are crucial. However, these components occupy a considerable amount of space and require a complex setup to function effectively. The article [98] provides an insightful overview of different light source technologies for NPB. To resolve this problem, plasmonic light sources offer a promising solution.

Plasmonic light sources can provide a compact alternative and can generate the required wave through the amplification of surface plasmons within cavities [99]. Using a silica spacer, Jimenez et al. in [100] coupled an electrically biased vertical cavity LED (VLED) with a plasmonic gold thin film, and a gold nanograting was placed above the thin film to propagate the far-field plasmonic wave. Microfluidic channels were incorporated into the design to enhance delivery. In spite of this, the inefficient coupling between the VLED source and the plasmonic gold nanograting reduced sensitivity compared to other devices at about 3 × 10^−4^ RIU. In comparison with other PL-pumped SPR devices, it has weaker signal-to-noise ratios. As a result of significant absorption of the propagating light, the VLED body material hinders testing with real biological analytes. S-polarized modes generated noise and obscured the response of p-polarized modes coupled to the plasmonic sensor, which was another challenge.

Quantum dot LEDs, which are LEDs that use quantum dots as the light-emitting material, were used as an integrated light source in [78]. In this study, an Al-ITO-Al (MIM) pattern was used to enhance the intensity of the generated light waves in a normal direction to the grating surface (see Figure 3C). In this design, coupling the laser signal to the on-chip waveguide without substantial loss is the primary challenge [99]. According to [101], up to 60% of the source could be coupled to the waveguide efficiently.

#### 5.2.2. Waveguide and Optical Fibers

Prisms are used in portable Kretschmann SPR biosensors to compensate for the discrepancy between the wave number of the plasmonic wave and the incident wave. This type of coupling delivers an incredibly sensitive sensing mechanism. Prisms, however, impede biosensor miniaturization for point-of-care applications [102]. To overcome this challenge, alternative coupling methods such as waveguides and optical fibers can be used instead of prisms, thereby reducing the biosensor’s size and cost while providing efficient wave localization [103,104].

**Figure 4:**
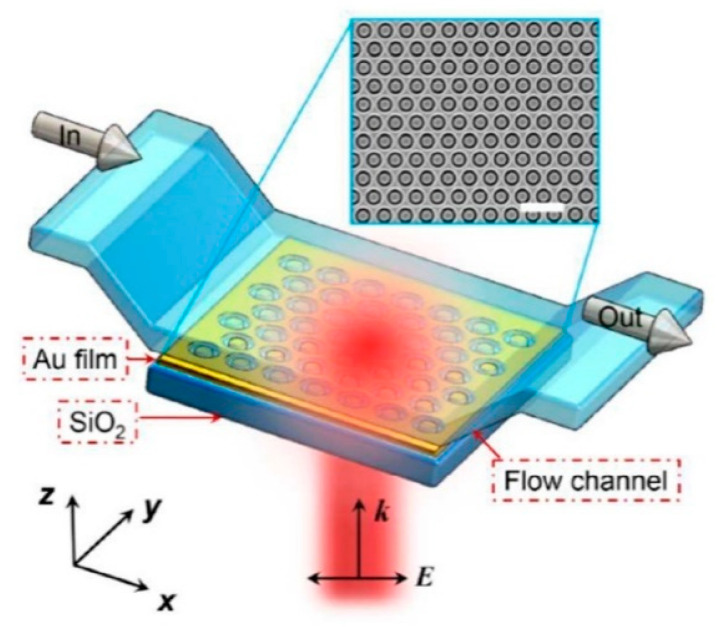
Miniaturized plasmonic nano-ring resonator sensor device with a fluid flow channel. Reprinted with permission from [105]. Copyright 2017 American Chemical Society.

In [105], a plasmonic nano-ring resonator is incorporated into an optical fiber to demonstrate the integration of a plasmonic sensor with an optical fiber (see Figure 4). Among the challenges of this design are the incorporation of an adhesion Ti layer between the sensor and the fiber, as well as the broadening of the plasmonic resonance when the numerical aperture of the fiber is large. A dielectric waveguide is used to connect the plasmonic resonator to an optical fiber in [104]. CMOS fabrication technology is fully integrated into the system with silicon-on-insulator (SOI) technology. Although this method offers many theoretical advantages, there is no experimental data to support it.

The study [83] implemented a polymer-integrated biosensing system (see Figure 3E). To function as a light source, a polymer light-emitting diode (PLED) was spin-coated onto a glass substrate. A photoluminescent (PL) material was deposited on the substrate’s opposite side to evanescently couple the transmitted light wave to the tantalum pentoxide waveguide. A coupling efficiency of 31% was found between the waveguide and the PL. Nevertheless, it is challenging to completely remove analyte residuals from the fluidic channels, which affects repeatability.

Using a complementary metal-oxide-semiconductor (CMOS) chip for imaging the beam transmitted from the plasmonic chip, the authors in [106] presented a battery-powered, handheld plasmonic biosensing system. Rather than using a lens to focus the beam, phase recovery was used to reduce the distance between the imager and the plasmonic chip. A gold array of nanoholes was used for high-throughput multiplexed sensing, but the device exhibited a high detection limit. Sensing devices should have a low detection limit to achieve high sensitivity. Several modifications were proposed to address this problem: replacing the LED with a laser source at the expense of a larger setup, improving plasmonic resonance sharpness, acquiring multiple images for different LED wavelengths, and using improved CMOS imaging chips with cooling circuits.

#### 5.2.3. Photodetectors

The final step of the biosensing process is the detection step, where the changes that result from the analyte interrogation upon the incident wave are observed by a photodetector or an imaging device, such as a camera with high responsiveness in the operating range of interest [107]. Real-time monitoring is possible with charge-coupled device (CCD) cameras. However, since the passivation layer consists of either silicon nitride or silicon dioxide, it may lead to lower sensitivity values due to their reflectance and absorbance profiles. A second challenge was the defects introduced in the photodetector during the fabrication process when the CMOS chip was exposed to the e-beam used to pattern the gold array [108]. There is also a weak overlap between the preferred plasmonic resonance range of gold (>700 nm) and the peak detection range of the photodetector (around 600 nm) [109].

A spectrometer with a wide wavelength range is required for spectral analysis of plasmonic resonance shifts. To track the optical response accurately, many spectrometers are required, which is a challenge for miniaturization. This challenge can be addressed with signal-processing techniques such as principal component analysis (PCA), which identifies the directions of maximum variation in data and maps the data to these directions. In [110], PCA was used to classify analyte gasses of a plasmonic gas sensor into four categories. In [111], it was used to analyze the Raman spectra of gold and silver nanoparticles. It was also used in [112] to remove higher-order components from the spectral response of plasmonic gold NPs assembled on DNA strands. Using plasmonic gold nanorods for gas sensing, PCA was used to reduce the number of spectrometers needed in [113]. The design becomes more compact by replacing the full spectrum analysis with a few wavelengths. Instead of using an external light source, the authors proposed harnessing thermal energy in combustion locations to excite the plasmonic sensor, which would minimize the sensor’s size and facilitate design integration. However, due to the low signal-to-noise ratio, the PCA was unable to provide adequate differentiation for the thermal imaging data.

An imaging objective and a plasmonic gold nanodisk sensor are integrated simultaneously in [114] by integrating a gradient index (GRIN) lens with a microfluidic channel. Signals reflected from the plasmonic sensor appear as parallel rays from the GRIN lens, making coupling to a spectrometer easier. Due to the absence of an adhesion layer, the lift-off step during electron beam lithography (EBL) introduces defects into this design. Additionally, the pinhole used to focus the reflected signal increases the device’s size.

Smartphone cameras for imaging, software applications for data processing, and phone batteries for powering the sensor are being used as emerging tools for the integration of light detectors [26]. This approach reduces the size of the biosensor and speeds up the sensing process. Ref. [115] provides a comprehensive overview of the use of smartphones for SPR measurements. Smartphone imaging is challenged by its limited resolution [26,115], which may improve with time as smartphone camera technologies advance.

### 5.3. Flexible NPB

For further development of portable NPBs, the use of flexible materials has been a bright spot for the field. Flexible NPBs combine flexible substrates with plasmonic materials for the detection of biological molecules. This flexibility allows the sensors to conform to various shapes, which makes them suitable for continuous monitoring, integration with soft tissues, and wearable applications. One of their main advantages includes the ability to provide real-time, continuous monitoring in dynamic environments due to their adaptability to differing surface conditions. Additionally, their lightweight and bendable nature allows for more user comfort when considering point-of-care applications such as non-invasive diagnostics [116].

Chowdhury et al., in [117], proposed a wearable SERS-based sensor, offering flexibility, stretchability, and reliable performance under bending and stretching, using PDMS substrate. This design showed an EF of 10^11^, a scattering to absorption ratio of about 2.5, and a large hotspot volume of 40 nm × 40 nm × 5 nm. In addition, Wang et al., in [89], developed a wearable plasmonic-electronic sensor with “universal” molecular recognition, using a flexible surface-SERS metasurface to noninvasively extract and analyze multiple body analytes. This design maintained its plasmonic activity under deformation and successfully tracks trace amounts of drugs, offering a sensitive platform for real-time monitoring of drug metabolism. Additionally, ref. [86] presented a flexible, wearable substrate called SF-AAO-Au, combining silk fibroin, anodic aluminum oxide, and gold nanoparticles to create a biocompatible, highly sensitive SERS biosensor for glucose detection. In their study [90], Chung et al. introduced a flexible, nanofibrous SERS substrate made from thermoplastic polyurethane and gold coating for non-invasive sweat pH sensing, using pH-responsive molecules 4-MBA and 4-MPy. The results of the study demonstrated high resolution, fast sweat absorption, good repeatability, and stable performance over 35 days. Han et al. [92] developed a wearable sweat sensor combining Janus fabric for sweat collection and grapefruit optical fibers embedded with silver nanoparticles for sensitive SERS analysis of sodium lactate and urea. As can be concluded from the literature, the main challenge in designing future flexible NPBs will be to maintain the performance of flexible biosensors under deformation during stretching and bending.

### 5.4. Other Wearable SERS-Based Biosensors

Recent research has addressed the challenge of integrating SERS technology with wearable biosensors. Many of these designs have been using sweat as a sample of body fluids for continuous health monitoring. For example, Atta et al. [81] presented a wearable patch based on SERS that enables the simultaneous detection of lactate, urea, and glucose in sweat using gold nanostars on adhesive tape. This patch demonstrated high sensitivity, with LODs of 0.7, 0.6 and 0.7 µM for lactate, urea, and glucose, respectively. In another study by Zhu et al. [84], a wearable SERS sensor was introduced which was inspired by the omnidirectional light-harvesting of the compound eye, using an omnidirectional plasmonic nanovoids array (OPNA) to enhance detection stability during movement. This sensor features a robust structure that protects “hotspots” and an asymmetrical super-hydrophilic surface for concentrating analytes from sweat.

In addition to analyzing sweat, wearable NPBs can also be used to analyze other biological fluids. Xiao et al., in [85], presented a wearable diaper sensor that uses a handheld Raman spectrometer and SERS to non-invasively detect biomolecules like urea, creatinine, and bilirubin in urine. This sensor utilized a raspberry-shaped Au substrate for enhanced signal detection and higher sensitivity. Additionally, in the study [118], Xing et al. created a multifunctional wearable sensor using plasma-functionalized silk fabric coated with silver nanoparticles (SFs@Ag) to enable both biomechanical and biomolecular sensing. The sensor accurately detects mechanical pressure and biomolecules, supported by machine learning for recognizing muscle strain, and offers highly sensitive SERS detection for comprehensive personalized health monitoring, including potential applications in neuromuscular disorder diagnosis.

### 5.5. Microfluidics NPBs

The advantages of NPBs with microfluidic channels and reservoirs are numerous. As a result of microfluidic channels, analyte solutions are delivered to the plasmonic sensing surface more efficiently, increasing the reaction rate and reducing diffusion times [14,103,119]. Additionally, they reduce the number of analytes needed for accurate sensing [120]. Microfluidic channels enable multiplexed sensing [121], which increases chip throughput by delivering analytes to multiple sensing spots at once [74,122]. In [123], for instance, 264 sample analytes are processed simultaneously in a single experiment. By using microvalves and row multiplexer gates, microfluidic flow is precisely controlled. The entire sensing area measures 2.5 cm x 2.5 cm. Moreover, microfluidic chambers are used to trap and incubate cells [124,125], ensuring that the analytes are located near the detection sites. As a result, the sensing process is enhanced, and the LOD is lowered [126].

The most common material used to fabricate microfluidic channels is PDMS, but alternative materials such as PMMA [34,74] and paper [127] have also been used. The integration of PDMS with metallic nanoparticles (NPs) provides a stretchable substrate with tunable properties [127], which provides advanced analyte manipulation and operational flexibility. The hydrophobicity of PDMS makes it challenging to integrate plasmonic sensors with microfluidics [128]. As a result, plasmonic NPs will have weak binding to PDMS channel walls. In this way, they can easily dissolve in the analyte solution flowing through the channel. This issue should be addressed by surface modification [129]. For plasmonic biosensing systems to be small-footprint and high-performance, these components must be integrated onto a substrate.

Microfluidics can also be introduced into paper-based biosensors for better real-time monitoring. Li et al. [91] introduced a flexible, wearable paper-based microfluidic device using SERS for continuous, real-time monitoring of uric acid and pH. Another study created a wearable microfluidic SERS-based NPB capable of the non-invasive detection of biomarkers such as urea, lactate, and pH in sweat. This design offers a miniature, thin plasmonic metasurface with homogeneous mushroom-shaped hot spots, providing high-resolution analysis by preventing the mixing of old and new sweat [87]. Mogera et al. [88] introduced a wearable plasmonic paper-based microfluidic system for continuous, simultaneous monitoring of sweat loss, sweat rate, and metabolites using label-free SERS to detect uric acid. Overall, wearable designs based on microfluidic technology have demonstrated substantial potential for continuous and long-term monitoring of pH, lactate, and uric acid levels.

## 6. Strategies for Enhancing Reproducibility

Recently, several studies have focused on investigating strategies to enhance the reproducibility and reliability of NPBs. One of the major obstacles in nanoplasmonic biosensors is the fabrication of uniform, reproducible nanostructures, because nanostructure size, shape, and spacing can significantly impact their biosensing performance [130]. One key approach is the precise control and optimization of the design of nanostructures and the implementation of advanced nanofabrication techniques.

Advances in nanofabrication methods have further enhanced the reproducibility of NPBs. For instance, methods such as block copolymer micelle nanolithography have been employed to produce gold nanoparticle arrays, significantly enhancing the reliability of plasmonic biosensors. This technique offers precise control of the size and density of the nanoparticles while being cost-effective and easily scalable [131]. Another approach is the integration with on-chip reference sensors and microfluidics, which led to the emergence of Lab-on-chip optical biosensor platforms. For example, utilizing patterned plasmonic nanostructures within microfluidics has displayed great potential in label-free, multiplex ultrasensitive detection while enhancing the uniformity of the sensors [26,132,133]. This platform minimizes sensor-to-sensor variation by automating sample handling and measurements. Moreover, on-chip reference sensors have been shown to correct non-specific effects and environmental fluctuations and improve the statistical reliability of measurements.

Furthermore, researchers have explored different strategies to optimize surface chemistry and biofunctionalization techniques. Self-assembling techniques for fabricating pillar arrays spiked with nanoparticles and self-assembled monolayers have been documented to generate high-density hotspots and reproducible functional surfaces across batches [134,135,136]. Employing stable bioconjugation procedures such as covalent immobilization or biotin-streptavidin surface functionalization has proven to improve the consistency of bioreceptors on the plasmonic surface, thereby enhancing the reproducibility of the biosensors [137].

An additional approach involves the integration of plasmonic nanoparticles with other nanomaterials, such as porous silicon or fluorescent quantum dots, to create novel hybrid materials. These integrated systems have proven to demonstrate increased sensitivity and reliability as a result of the materials’ synergetic properties [133,138,139]. For example, hybrid materials containing porous silicon have been illustrated to have improved surface biocompatibility and tunability. These properties are critical for maintaining reliable sensor performance [138].

Recent progress in machine learning and data processing technologies is also aiding in improving the reproducibility of plasmonic biosensors. The capacity to process datasets produced by these sensors instantaneously can result in calibration and error adjustment procedures, which consequently enhance the reliability of the recorded measurements [140]. As an example, portable biosensing devices, when connected to gadgets, can enable instantaneous responses and modifications to counter the challenges associated with environmental changes during measurements [140,141,142].

**Figure 5:**
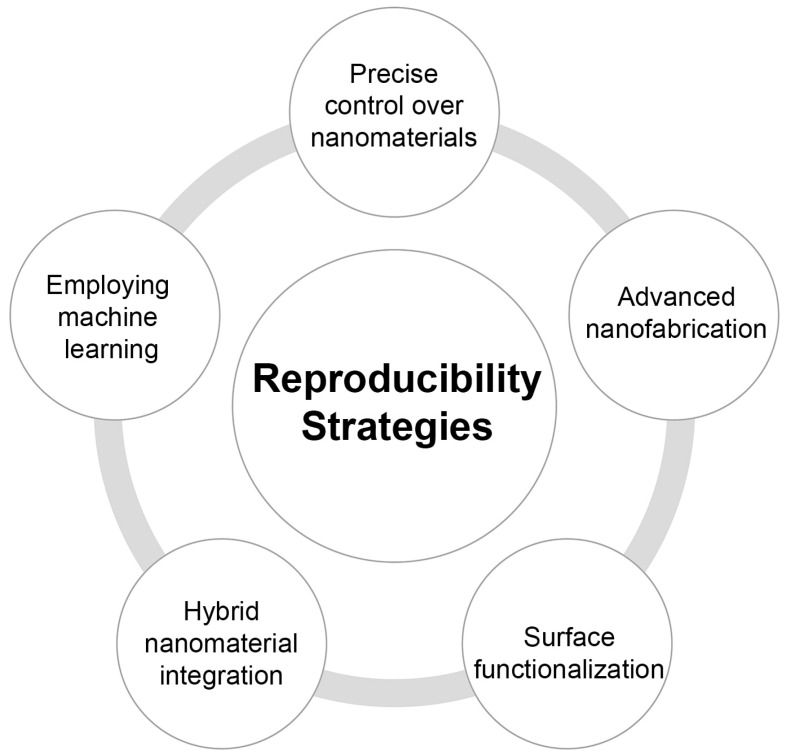
The schematic of strategies to enhance reproducibility of NPBs.

To summarize, enhancing the reproducibility of plasmonic biosensors can be realized by precise nanostructure control, advanced nanofabrication techniques, surface functionalization techniques, hybrid nanomaterial integration, and employing machine learning for data analysis and interpretation (Figure 5). Not only do these approaches tackle the issues of inconsistency, but they also open up opportunities for the wider use of plasmonic biosensors in various fields, including medical testing, healthcare monitoring, and environmental uses.

## 7. Applications

**Figure 6:**
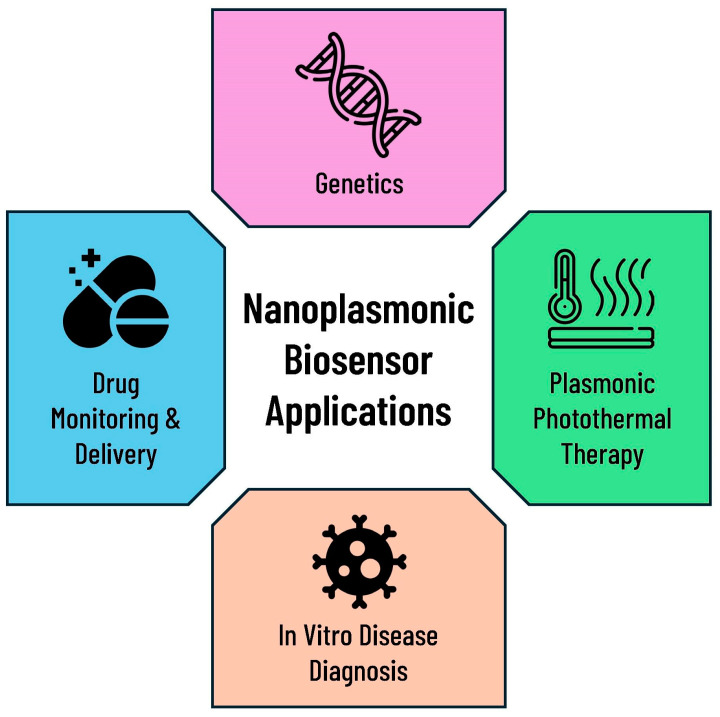
Potential applications of nanoplasmonic biosensors.

Many attempts have been made to demonstrate the potential of plasmonic sensors. A growing number of applications of NPBs have attracted special attention in recent years, including in point-of-care diagnosis and prognosis [19], healthcare monitoring [143], and early cancer diagnosis [144]. The field has demonstrated significant advancements and applications across not only biomedical applications but also other fields, including environmental pollution evaluation [145], water-borne virus detection [146,147], food safety [148], detecting biological and chemical threat agents [149], multispectral imaging devices [150,151,152], plasmonic fiber-optic sensors [153,154,155], and solar cells [156,157]. A number of promising results have been obtained by utilizing plasmonic sensors for the detection of environmental pollutants, such as pesticides, toxic heavy metals, and microorganisms, which have been released to the environment either in a controlled or uncontrolled manner [158,159]. Additionally, these sensors could have been utilized for the routine testing of contaminants in food and water for the purposes of ensuring food safety and quality [160,161]. Global concerns, therefore, will be addressed through recent technological advancements [7]. As shown in Figure 6, all applications are classified into four groups, which will be reviewed in the following section.

### 7.1. In Vitro Disease Diagnosis

The NPBs designed for in vitro diagnosis are able to provide high-accuracy measurement over a wide range of concentrations, which is extremely beneficial for clinical diagnosis. To monitor and diagnose diseases, a wide range of chemicals, target biomolecules, disease biomarkers, antibodies, proteins, nucleic acids, and enzymes have been addressed in the plasmonic literature [162,163]. Owing to the fact that the diagnosis of many cancer types at early stages expedites their treatment, utilizing a plasmonic biosensor for diagnosing cancer’s biomarkers, including Neuron-Specific Enolase, microRNA, and Cytokines, is an emerging approach [164]. The performance of the biosensor, therefore, has been investigated for its ability to detect various cancer markers, including human epidermal growth factor receptor 2 (HER2), doxorubicin (DOX), alpha-fetoprotein (AFP), Carcinoembryonic antigen (CEA), C-reactive protein, and prostate-specific antigen (PSA) [14,21,165]. The two latter biomarkers have been evaluated for prostate cancer diagnosis as an antigen [166]. They can also be used for cancer drug screening, namely Mucin-1 Anticancer drug.

Using NPBs to measure biomarkers associated with neurodegenerative diseases demonstrated promising results for future POCT devices in this field of research. A few studies have been conducted in this field to measure Alzheimer’s’ chemical biomarkers concentrations, namely amyloid beta peptides and tau proteins, in patients’ blood serum and cerebrospinal fluid [167,168,169]. Other analytes, including NaCl, D-glucose, Adenine, Thiram, 4-amino-benzoic acid, Pyrocatechol, Tobramycin, Thrombin, Lectin, CA125, HE4, Eotaxin-1, and PTX3, have also been measured via plasmonic nano-biosensors [14]. In addition, the detection of cytokines, signaling proteins of inflammation in the human body, namely IgE, IgG, Interleukin-1 (IL-1), IL-2, IL-4, IL-6, IL-10, Interferon gamma (IFN-γ), Tumor Necrosis Factor alpha (TNF-α), and matrix metalloproteinase-3 (MMP-3) enzyme, have been addressed in the literature [14,170].

### 7.2. Plasmonic Photothermal Therapy

SPR technology was used for not only biosensing applications but also cancer therapy, viz. plasmonic photothermal therapy (PPTT) [171]. Recently, a method has emerged which utilizes the unique photothermal properties of gold and silver nanoparticles to convert light into heat, corresponding to the LSPR absorption band, and selectively target cancer cells, a process that occurs rapidly. The photoexcitation of the metal nanoparticles induces the formation of a heated electron gas that cools rapidly within 1 ps by exchanging energy with the lattice of the nanoparticles (NP). Within 100 ps, the NP exchanges energy with the surrounding medium through phonon–phonon interaction [172,173]. Gold and silver nanoparticles can be precisely tuned for biomolecular targeting to induce the death of cancer cells by precise heating [174]. Dr. El-Sayed’s group, in one of their seminal papers in 2008, demonstrated that in vivo PPTT could be used for treating deep-tissue malignancies with inexpensive near-infrared lasers and gold nanorods [175]. Recent studies have focused on two different areas of plasmonic photothermal therapy: the development of new nanostructures and the integration of cancer biosensors with PPTT.

Many attempts have been made to improve photothermal conversion efficiency in the last decade. Gold nanostars in PPTT have received particular attention due to their high heat conversion efficiency. Chatterjee et al. investigated the effects of sharpness on the enhanced field at the tip head and the temperature distribution localized there by using gold nanostars as a model structure [176]. A study conducted by Dr. El-Sayed and his colleagues in vitro determined the ideal size of gold nanorods (AuNRs) by comparing their plasmonic properties as well as their efficacy as photothermal contrast agents [177]. The amount of heat generated by the nanoparticles upon NIR laser irradiation depends on the amount of absorbance to total extinction, the electric field, and the distance from the nanoparticle surface [177]. The authors of [178] carried out a study using AuNRs to initiate apoptosis in different cell lines and compared the results. In all three cell lines, PPTT resulted in a significant reduction in viability and an increase in apoptosis [178]. A special black noble metal core–shell nanostructure has been reported by Liu et al. in [179], consisting of silver (Ag) nanocubes as the core, and amino acid-encoded, highly branched Au nanorods as the shells. Additionally, polydopamine-coated gold nanoclusters have shown excellent photothermal properties with a conversion efficiency of 60.3% and better stability [180]. Ayala-Orozco et al. in [181] compared 90 nm diameter Au nanomatryoshkas (Au/SiO_2_/Au) and 150 nm diameter Au nanoshells for the treatment of highly aggressive triple negative breast cancer (TNBC) tumors in mice. According to this study, Au nanomatryoshkas are more effective for PPTT because of their smaller size and larger absorption cross-section. Based on these findings, we can conclude that the efficiency of photothermal conversion varies depending on the shape, size, metal, and configuration of the nanostructure of the nanoparticle.

Besides advancements in novel nanostructures, biosensors have been integrated with therapeutic modality with the aim of developing a novel cancer treatment approach [182]. Recent literature reviews found that gold nanostars can be used not only for photothermal treatment, but also for tumor imaging and as supplemental treatment in immunotherapy, which has the ability to enhance overall cancer treatment efficacy [183]. The development of nanoparticle-based combination therapies further supports the concept of multimodal therapeutic approaches against tumors of various complexities [184]. Nair et al., in [185], reported the synthesis of a hybrid nanomaterial consisting of an extremely small gold nanocluster and an asymmetric gold nanostructure, a gold nanorod, with unique optical properties such as tunable emission and NIR absorption. The hybrid system was designed to emit and absorb in the NIR region so it could be used simultaneously for imaging and therapy [185]. Future cancer therapies may benefit from these innovative approaches.

### 7.3. Drug Monitoring and Delivery Systems

These biosensors can also be used to measure the concentration of drugs and molecules with clinical applications, namely antibiotics, anticonvulsants, and anticancer drugs [186]. This application is helpful for real-time pharmaceutical studies, which in turn aids in keeping therapeutic drugs within desired ranges. Ilkhani et al. developed an NPB, utilizing gold-coated Fe_2_Ni@Au nanoparticles functionalized with DNA and working based on the SERS/electrochemical transduction. The device was designed to measure DOX, a chemotherapeutic drug used in the treatment of breast cancer, as well as to assess DNA modification and dose dependence [187]. In a study by Muneer et al., gold nanostructures were deposited on a nickel foam substrate to detect a specific antibiotic, meropenem, in human blood plasma at picomolar concentrations [188]. The latter application of NPBs contributes to the mitigation of microbial resistance. The controlled release of medicines can be achieved by integrating NPBs with drug delivery systems. In this way, medicines, prescribed by doctors, are released at specified amounts and concentrations, thereby enhancing treatment efficacy and minimizing adverse effects [189]. Incorporating these technologies into drug delivery systems will improve personalized medicine [190].

### 7.4. Genetics

A number of recent studies have demonstrated that these biosensors can be used in gene monitoring, specific nucleic acid sequence detection, and gene expression studies [162]. Previous studies have demonstrated that plasmonic nanoprobes could be used for detecting specific DNA sequences associated with severe diseases, including HIV and breast cancer [191]. As reported by Ma et al. in [192], the LSPR biosensor is capable of detecting mutant DNA and telomerase, which are cancer biomarkers. LSPR nanoaptasensors, proposed by Tian et al., based on Au@Ag core–shell nanocubes, provided real-time monitoring of the formation process of G-quadruplex structures [193]. Nguyen et al. developed the LSPR biosensor to identify mutations and methylation of circulating tumor DNA of the PIK3CA gene, a biomarker for cancer assessment [194]. The method described by Crawford et al. is based on plasmonic probes for the real-time imaging and biosensing of mRNA biomarkers in plant leaves [195]. These studies have showcased the impressive potential of NPBs in genetics.

## 8. Challenges and Trends

Research papers on NPBs have recently followed specific lines of scientific investigation. These challenges and trends are classified into five groups, which are performance metrics, biorecognition, practical constraints, surface chemistry, and integration with emerging technologies. In the first group, researchers try to improve performance metrics of biosensors, including sensitivity, LOD, FOM, and specificity, using innovative synthesis and design techniques. The second line of research focuses on finding new biological or synthetic materials to detect specific chemical biomarkers, proteins, mRNA, etc. Practical constraints include high cost, bulkiness, long-term stability, and the level to which a material is prone to environmental conditions, which will be explained here. Another issue in this field is the distinction between surface and bulk sensitivities. In addition, several new emerging technologies, namely artificial intelligence (AI), Information and Communication Technology (ICT), neuroscience, etc., have been integrated into these biosensors; however, serious challenges still need to be addressed.

### 8.1. Enhancing Performance Metrics

While the results of recent studies have notably improved measurement parameters, namely sensitivity, LOD, FOM, etc., of these biosensors, more research is required to achieve perfect technology [196]. Recent research has focused on improving biosensors’ LOD and sensitivity through strategies such as optimizing nanostructure design, material selection, and modifying sensor interfaces [44,197]. The use of advanced sensing techniques, such as refractometric sensing, plasmon rulers, and chiral plasmonics, has also been studied to lower the LOD [197]. An approach developed by Facson et al. in [198] involved using metallic labels to amplify signals. According to [199], the main issue with commercial SPR biosensors is their limited LODs due to noise levels, which generally range between 10^−6^ and 10^−5^ RIU. Caucheteur et al. [200] reported that gold coatings and roughness significantly affect the sensitivity of plasmonic fiber gratings for the detection of a cancer biomarker, cytokeratin 17. While these advancements in NPB technology are pushing the LOD towards single-molecule detection, more studies are needed.

In terms of high-sensitivity detection, several strategies have been proposed in particular. These strategies include optimizing nanostructure design, such as using nanowires or nanogrooves to amplify plasmonic fields [201,202], and employing nanoparticle-antibody conjugates to amplify signal shifts [203]. In addition, experimental approaches involve fabricating plasmonic array nanostructures and site-selective immobilization, while mathematical approaches utilize LSPR inflection points to improve refractive index sensitivity [204]. Other sensitivity-enhancing strategies include signal amplification through polymerization and nanocatalysts, diffusion-limit-breaking systems, and combined approaches utilizing renal concentration and chemical signal amplification [205]. There have also been several studies addressing the issue of achieving high FOM. Phase-sensitive measurements have shown potential in boosting FOM by reducing linewidths [206]. Optimization strategies include coupling LSPR with other phenomena, such as Wood’s anomaly or surface lattice resonances, to enhance sensitivity and FOM [207]. In summary, although NPB performance is approaching its theoretical limits as a result of these advancements, further research is required.

LSPR sensors are prone to interference from non-specific binding and biofouling in complex matrices [199,208]. There have been several strategies developed for addressing this challenge, including self-assembled monolayers, polymer brushes, and nanopatterned surfaces [209,210,211]. Vasocherová et al. [211] have studied functionalized ultra-low fouling carboxy- and hydroxy-functional surfaces. In addition, Jiang et al. 2020 [212] reported antifouling sensors that can operate in complex sample matrices such as serum. Lane et al. [213], presented a tool for designing liquid media systems that modulate specific intermolecular interactions to reduce nonspecific binding in detection technologies, using reagents like thiocyanate ions and glycylglycylglycine. Despite progress, challenges remain in developing stable antifouling techniques for real-life samples.

### 8.2. Biorecognition

While plasmonic nanoparticles can be used as label-free optical sensors by attaching molecular recognition elements to the nanostructures, it is unclear whether and how functionalization of nanostructure hotspots will maximize sensing efficiency. Recent research has focused on optimizing the functionalization of hotspot regions of nanostructures. Various methods have been developed, including site-specific passivation [214], UV-laser interference lithography [215], and nano-localized water heating [216]. Goerlitzer et al. in [214] presented a high-throughput method for the site-specific functionalization of NPBs using crescent-shaped nanostructures, which achieved efficiency of 90% for thiol molecule detection. UV-Laser interference lithography has been introduced in [215] by Quilis et al. for the local functionalization of plasmonic hotspots on gold nanoparticles. The authors created periodic arrays using a UV-induced thermoresponsive poly(N-isopropylacrylamide)-based (pNIPAAm) hydrogel matrix. The rapid ‘bottom-up’ approach uses pulsed laser irradiation to heat the surrounding water to functionalize selective regions of plasmonic nanostructures, as proposed by Jack et al. [216]. In [217], using a rotating coordinate system in colloidal lithography, Yang et al. created a hierarchical plasmonic nanostructure with enhanced hot spot density and tunable LSPR. Heterodimeric plasmonic nanogaps formed between gold nanostars and nanospheres showed high sensitivity for detecting single protein molecules and nucleic acid fragments (Chatterjee et al.) [218]. In spite of advances made in functionalizing nanostructure hotspots, more research is needed to produce stable, widely applicable, cost-effective, scalable, and easy-to-use biosensors in the near future.

### 8.3. Practical Constraints

The high cost and bulky size of SPR instruments prevent their commercialization and use at point-of-care [98]. Recent efforts have focused on miniaturizing and reducing the cost of SPR and LSPR devices. Approaches include using alternative light sources like LEDs and smartphone displays [98], integrating LSPR with microfluidics [74], and developing portable platforms [96]. In addition, combining light sources with waveguide-based plasmonic devices can reduce both the sizes and costs of NPBs [219]. Besides cost and size limitations, power supply for light sources, efficient coupling between integrated light sources and waveguides, and reduction in SPR losses are the three other major challenges in actual applications [219]. According to [199], it is not practical to carry out point-of-care testing with phase modulation SPR systems. This is because their optical configurations are very complicated. Therefore, future works should focus on the cost, size, portability, and ease-of-use of this technology, as well as using semiconductor dielectric materials such as silicon or germanium to mimic plasmonic metals while minimizing heat losses. Future research will also focus on nanopattern arrays composed of microvalves and distributed channels.

Other major challenges to commercializing LSPR include the long-term stability of transducers, multiplex sensing, and high-throughput production [220]. In the study [221] by Wang and Tang, it was demonstrated that gold nanorod arrays can be used to create multiplex biosensors for simultaneous multi-sample analysis, with distinct plasmon peaks corresponding to different analytes. The results of the study demonstrated linear sensitivity for all targets with minimal cross-reactivity. In addition, Aćimović et al. [222] developed an NPB for the real-time detection of cancer biomarkers in complex matrices with high sensitivity, using 32 sensing sites and eight independent microfluidic channels. According to Xiang et al. [223], to develop cost-effective, high-throughput LSPR biosensors, large-area fabrication techniques, such as nanosphere lithography, are essential, which should be addressed in future research.

In future works, flexible and wearable NPBs can be developed by selecting a proper polymer substrate based on factors including wettability, surface roughness, thermal stability, and biocompatibility. These flexible NPBs should be designed to withstand bending, stretching, and twisting. Energy supply considerations are also rarely addressed for NPBs in point-of-care applications. It is essential that self-powered NPBs provide or harvest power for light sources, photodetectors, microfluidic actuators for the manipulation of analytes, and electronic circuitry for signal processing. It will also be possible to integrate metallic nanoparticles with 2D semiconductor sheets in future NPBs, thus combining the plasmonic properties of metal with the tunability and ultra-thinness provided by 2D semiconductor sheets. Further research is also required to design plasmonic components that deliver robust performance under harsh industrial conditions. Furthermore, future research will need to address the effective removal of analytes post-sensing, namely reusability, LOD dependence on the amount of sensed analyte, and reproducibility of bottom-up synthesized nanostructures. Therefore, there are still many challenges that need to be overcome in order to improve practical applications of NPBs.

### 8.4. Surface Chemistry

In most cases, NPBs are evaluated by immersing the nanostructures in liquid samples and observing the peak shifts, which are referred to bulk sensitivity. For this sensitivity, average electromagnetic fields close to whole plasmonic nanostructures are taken into account. On the contrary, surface sensitivity is related to a confined region of the electromagnetic fields around them [224,225]. Li et al., in [226], compared the surface and bulk sensitivity of diffractively coupled plasmonic crystals using atomic Al_2_O_3_ layers. It has been shown that, as opposed to the bulk sensitivity, the surface sensitivity shows an opposite dependence on coupling strength and metal thickness. It was also demonstrated in [225] that optimizing interparticle gaps in nanoparticle dimers can significantly enhance surface sensitivity compared to bulk sensitivity. Ricciardi et al. [227] demonstrated that there is a significant difference between bulk and surface sensitivities in NPBs based on Rayleigh anomalies. Furthermore, thin overlays of analytes decreased surface sensitivity by two orders of magnitude compared to bulk sensitivity. Overall, the difference between bulk and surface sensitivities when designing NPBs should be considered in future works.

### 8.5. Emerging Technologies

Miniaturizing NPBs for Lab-on-chip devices and integrating them with portable technologies, such as microfluidics, can be challenging [228]. Integrating LSPR sensing technology with microfluidics enables automation, precise control, and enhanced sensing efficiency by using small volumes and rapid processing [74]. Microfluidics also improves reaction rates, reduces diffusion times, and supports efficient surface regeneration [74]. In addition, the integration of NPBs with microfluidics enhances multiplexing capabilities and promotes high-throughput analysis [132]. Aćimović et al. [222] have demonstrated the parallel, real-time detection of cancer biomarkers in complex matrices using LSPR chips with integrated microfluidics. In terms of improving sensitivity, the integration of gold bipyramidal nanostructures into microfluidic devices has demonstrated bulk refractive index sensitivities of up to 243 nm RIU^−1^, according to Campu et al. [229]. Microfluidic designs offer insights beyond traditional transduction methods into various phenomena at liquid–liquid and solid–liquid interfaces [230]. Bhalla and Shen [230] have also applied LSPR and microfluidics to monitor DNA polymerase activity and analyze biological fluids. Despite considerable progress, further research is needed in plasmonic-based microfluidics.

To the best of the authors’ knowledge, the integration of NPBs with Information and Communication Technology (ICT) has not been addressed in the literature [231]. Similarly, integrating artificial intelligence (AI) and machine learning techniques with NPB technologies has rarely been studied. Machine learning approaches can improve data processing, sensor accuracy and reliability, and signal-to-noise ratio [232]. In addition to analyzing large sets of data, such approaches can also correlate data and physiological events, even from noisy and low-resolution sensors. Mortazavi et al. used neural networks to model LSPR nanoparticles, which allows for the prediction of device characteristics based on the dimensions of the nanoparticles [233]. Dong et al. [234] developed AI-assisted signal processing algorithms to improve sensor specificity and sensitivity. Liang et al. [232] developed biosensors based on NPBs and AI methods that can detect SARS-CoV-2 virus particles directly. The results of the study indicate that the viral concentration can be detected with an accuracy greater than 97% and a detection limit of 100 vp/mL within 12 min in the range of 125.28–10⁶ vp/mL. The integration of AI and LSPR technology, therefore, paves the way for improved design, data analysis, and performance.

## 9. Conclusions

In light of a comprehensive review of the literature, we have concluded that NPBs hold huge potential for single biomolecule detection in POCT devices owing to their simplicity and cost-effectiveness as well as other excellent intrinsic features, namely their ultra-high sensitivity, low noise background, real-time monitoring, high reusability, small molecules, and label-free detection. A wide range of applications have also been developed in the field of NPBs, such as disease diagnosis, photothermal therapy, genetics, and drug monitoring and delivery. For all their potential applications and benefits, NPBs face several technical challenges that are to be addressed to allow high throughput production and usage.

According to the review of the literature, other dielectric function models have been reported, in addition to the Drude and Drude-Lorentz models, two of the most commonly used models. These models include the Brendel-Bormann, multiple oscillators, and Pade-based generalized dispersive material models, which predict dielectric properties with higher accuracy and for wider spectral ranges. The dielectric function model has also been shown to be more accurate when re-tuned for specific metals and wavelengths. Despite progress in the field, more research is needed to develop models for all spectral ranges and nanomaterials.

A recent development in plasmonic nanomaterials has contributed to advancing the performance of biosensors. Metals such as Au, Ag, and Cu, are generally suitable plasmonic materials due to their abundance of free electrons. However, they exhibit certain drawbacks, including limited chemical stability, high ohmic losses, and non-tunable conductivity. In contrast to metals, semiconductors, including aluminum-doped zinc oxide and indium-doped tin oxide, can be tuned according to the size, shape, and composition of the nanoparticles. This tunability enables the development of highly sensitive and selective biosensors. As a result of further advances in 2D nanomaterials, not least graphene, NPBs have achieved superior success in not only sensitivity but also selectivity. They are ideal for portable devices with light sources of limited wavelength ranges since they operate between terahertz and mid-infrared frequencies. This progress in plasmonic nanomaterials indicates that, by using 2D nanomaterials’ unique properties in combination with novel fabrication techniques, researchers will design next-generation portable NPBs with outstanding performance metrics.

In recent years, portable NPBs have gained popularity for rapid and on-site medical testing, especially in remote areas, by offering quick, low-cost, and user-friendly performance. The literature review indicates that portable NPBs can be divided into five groups, namely portable SPR, miniaturized components, flexible NPBs, wearable SRS-based designs, and microfluidic NPBs. The miniaturization of optical components has been addressed in recent studies, since portable SPR designs require all components to be small. In addition to portable SPR designs, flexible NPBs use lightweight, conformable, flexible substrates and plasmonic nanomaterials, making them ideal for wearable and continuous monitoring. However, maintaining sensor performance during stretching and bending remains a challenge. SERS technology has also been integrated with wearable biosensors to monitor health conditions continuously, using sweat as a sample of body fluids. Biological fluids other than sweat have also been analyzed by wearable SERS-based NPB. In addition to these designs, microfluidic-based NPBs offer several benefits, such as improved sensing efficiency, reduced analyte requirements, and multiplexed sensing for higher throughput. The weak binding of Plasmonic NPs to PDMS channels in these types of NPBs allows them to dissolve in analyte solutions, which requires further surface modification. Future portable NPBs will address key challenges, including the miniaturization of components, maintaining sensor performance during deformation, and addressing nanoparticle binding issues.

Reproducibility is one of the major hindrances to the commercialization of NPBs. In order to improve this parameter, researchers have found that it is possible to control nanostructures precisely, use advanced nanofabrication techniques, incorporate hybrid materials, and apply machine learning to data analysis. Indeed, in the design of the next-generation NPB, advanced reproducibility techniques for fabrication and performance will be considered.

There are five areas of upcoming trends in the NPB field of research, namely performance metrics, biorecognition, practical constraints, surface chemistry, and emerging technologies. Current advancements in NPBs have enhanced sensitivity and LOD, and pushed performance towards theoretical limits. Excellent progress has also been made in functionalizing plasmonic nanostructures, including site-specific passivation, UV-laser interference lithography, nano-localized water heating, and hierarchical plasmonic nanostructures. Nevertheless, challenges remain, including the need for more stable antifouling techniques for real-life samples and scalable, cost-effective biosensors. NPBs have also been limited in practical applications and commercialization due to their size and power requirements. Therefore, future research should focus on overcoming wearability, portability, flexibility, microfluidic-based NPBs, long-term stability, and reusability. Innovative technologies such as ICT, AI, and machine learning are integrating with NPBs to boost data analysis and performance.

## Figures and Tables

**Table 1:** Summary of plasmonic nanoparticles and nanostructures.

Class of Nanomaterials	Examples	Merit	Limitation
Metal nanoparticle	Au, Ag, Cu	superior plasmonic performance	chemical stability, high ohmic losses, non-tunable conductivity
Doped semiconductors	AZO, In−SnO_2_	chemical stability, tunability	moderate plasmonic performance
Dielectrics	TiN	chemical stability, tunability	moderate plasmonic performance
2D nanomaterials	Graphene	strong field confinement, high tunability	low plasmonic performance in visible spectrum, surface functionalization, edges effect
Advance nanostructures	MoS2/g-CN/AuNP	superior mechanical strength, higher heating rates, aggregation prevention	precise control over effective parameters

## Data Availability

The raw data supporting the conclusions of this article will be made available by the authors on request.
